# Dichlorido[(1*R*,2*R*)-*N*-(pyridin-2-yl­methyl)cyclo­hexane-1,2-diamine-κ^3^ *N*,*N*′,*N*′′]mercury(II)

**DOI:** 10.1107/S1600536812003340

**Published:** 2012-02-04

**Authors:** Chuan-Zhu Gao, Xiu-Ying Zhang, Lin Cheng

**Affiliations:** a Department of Chemistry and Chemical Engineering, Southeast University, Nanjing 211189, People’s Republic of China

## Abstract

In the title compound, [HgCl_2_(C_12_H_19_N_3_)], the Hg^II^ ion is coordinated by three N atoms of the (1*R*,2*R*)-*N*-(pyridin-2-ylmeth­yl)cyclo­hexane-1,2-diamine ligand and by a Cl atom in the basal plane, and by a second Cl atom in the apical position, within a distorted square-pyramidal geometry. The coordination of the enanti­opure ligand to the metal atom renders the central N atom chiral with an *S* configuration, so the complex is enanti­omerically pure and corresponds to the *S*,*R*,*R* diastereoisomer. Mol­ecules are linked *via* weak N—H⋯Cl hydrogen bonds into a one-dimensional hydrogen-bonding supramolecular chain along the crystallographic *b* axis.

## Related literature

For related structures, see: Cheng *et al.* (2011 ▶); Yin *et al.* (2011 ▶). For nonlinear optical applications and luminescence properties, see: He *et al.* (2010 ▶).
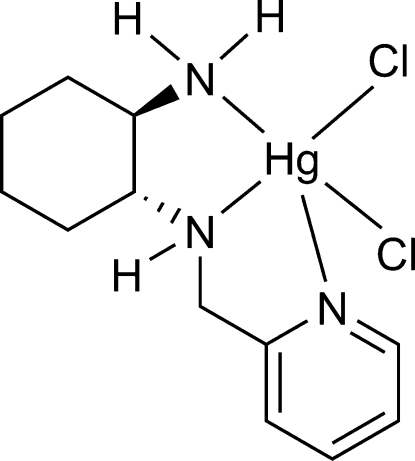


## Experimental

### 

#### Crystal data

[HgCl_2_(C_12_H_19_N_3_)]
*M* *_r_* = 476.79
Orthorhombic, 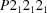

*a* = 8.5319 (12) Å
*b* = 8.8244 (12) Å
*c* = 19.688 (3) Å
*V* = 1482.3 (4) Å^3^
*Z* = 4
Mo *K*α radiation
μ = 10.73 mm^−1^
*T* = 123 K
0.08 × 0.06 × 0.06 mm

#### Data collection

Bruker SMART APEX CCD diffractometer
Absorption correction: multi-scan (*SADABS*; Bruker, 2000 ▶) *T*_min_ = 0.481, *T*_max_ = 0.565
11063 measured reflections
2902 independent reflections
2796 reflections with *I* > 2σ(*I*)
*R*_int_ = 0.028

#### Refinement

*R*[*F* ^2^ > 2σ(*F* ^2^)] = 0.021
*wR*(*F* ^2^) = 0.045
*S* = 1.06
2902 reflections
163 parameters
H-atom parameters constrained
Δρ_max_ = 1.08 e Å^−3^
Δρ_min_ = −0.68 e Å^−3^
Absolute structure: Flack (1983 ▶), 1206 Friedel pairs
Flack parameter: 0.009 (7)

### 

Data collection: *SMART* (Bruker, 2000 ▶); cell refinement: *SMART*; data reduction: *SAINT-Plus* (Bruker, 2000 ▶); program(s) used to solve structure: *SHELXTL* (Sheldrick, 2008 ▶); program(s) used to refine structure: *SHELXL97* (Sheldrick, 2008 ▶); molecular graphics: *PLATON* (Spek, 2009 ▶); software used to prepare material for publication: *SHELXTL*.

## Supplementary Material

Crystal structure: contains datablock(s) I, global. DOI: 10.1107/S1600536812003340/rk2329sup1.cif

Structure factors: contains datablock(s) I. DOI: 10.1107/S1600536812003340/rk2329Isup2.hkl

Additional supplementary materials:  crystallographic information; 3D view; checkCIF report

## Figures and Tables

**Table 1:** Hydrogen-bond geometry (Å, °)

*D*—H⋯*A*	*D*—H	H⋯*A*	*D*⋯*A*	*D*—H⋯*A*
N3—H3*B*⋯Cl2^i^	0.87	2.83	3.527 (5)	138
N3—H3*C*⋯Cl1^ii^	0.87	2.45	3.316 (5)	173

Symmetry codes: (i) 
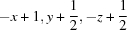
; (ii) 
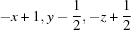
.

**Fig. 1.**
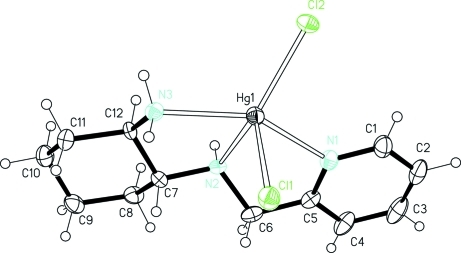
View of the title compound with the atom-labelling scheme. Displacement ellipsoids are drawn at the 30% probability level. H atoms are represented as small spheres of arbitrary radii.

**Fig. 2.**
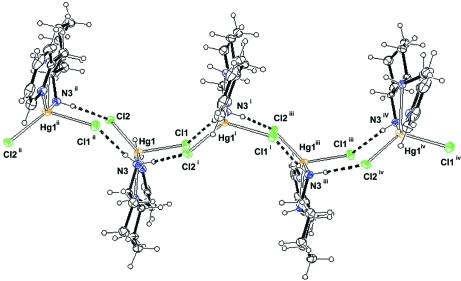
Partial packing view showing the weak N—H···Cl hydrogen bonding chain. Symmetry codes: (i) 1-x, 1/2+y, 1/2-z; (ii) 1-x, -1/2+y, 1/2-z; (iii) x, 1+y, z; (iv) 1-x, 3/2+y, 1/2-z

## supplementary crystallographic information

### Comment

Recently, there has been current significant interest in the rational design and synthesis of chiral coordination polymers due to their potential utility in enantiomerically selective catalysis and separations, second–order non–linear optical applications and luminescence (He *et al.* 2010). A basic design route for this kind of polymers is to appropriately organize the metal ions into ordered architectures by use of chiral ligands. Herein, we report a new chiral complex, Hg(2–*Amp*)Cl_2_, where 2–*Amp* = 2–(((1*R*,2*R*)–2–aminocyclohexylamino)methyl)phenol, as a chiral ligand.

The title compound is a mononuclear complex, in which the coordination environment of Hg^II^ can be described as distorted square–pyramidal, being surrounded by one tridentate ligand and two chlorine anions (Fig. 1).

The molecules are linked to each other, *via* weak N—H···Cl hydrogen bonds, into a one–dimensional hydrogen bonding network (Table 1, Fig. 2).

### Experimental

(1*R*,2*R*)-*N*^1^-(pyridin-2-yl-methyl)cyclohexane-1,2-diamine (0.041 g, 0.2 mmol) dissolved in water (8 ml) was added to a methanol solution (10 ml) of HgCl_2_ (0.054 g, 0.2 mmol). The mixture solution was stirred for 1 h at room temperature and then filtered. The filtrate was allowed to evaporate slowly at room temperature. After 2 weeks, colourless block crystals were obtained with 58.7% yield (0.056 g).

### Refinement

All H atoms attached to C atoms were fixed geometrically and treated as riding with C—H = 0.95–1.00Å with *U*_iso_(H) = 1.2*U*_eq_(C). H atoms attached to N atoms were located in difference Fourier maps and included in the subsequent refinement using restraints with N—H = 0.87Å with *U*_iso_(H) = 1.2*U*_eq_(N).

### Crystal data

**Table:**

[HgCl_2_(C_12_H_19_N_3_)]	*F*(000) = 904
*M**_r_* = 476.79	*D*_x_ = 2.136 Mg m^−^^3^
Orthorhombic, *P*2_1_2_1_2_1_	Mo *K*α radiation, λ = 0.71073 Å
Hall symbol: P 2ac 2ab	Cell parameters from 791 reflections
*a* = 8.5319 (12) Å	θ = 2.4–28.0°
*b* = 8.8244 (12) Å	µ = 10.73 mm^−^^1^
*c* = 19.688 (3) Å	*T* = 123 K
*V* = 1482.3 (4) Å^3^	Block, colourless
*Z* = 4	0.08 × 0.06 × 0.06 mm

### Data collection

**Table:**

Bruker SMART APEX CCD diffractometer	2902 independent reflections
Radiation source: fine–focus sealed tube	2796 reflections with *I* > 2σ(*I*)
Graphite monochromator	*R*_int_ = 0.028
φ and ω scans	θ_max_ = 26.0°, θ_min_ = 2.1°
Absorption correction: multi-scan (*SADABS*; Bruker, 2000)	*h* = −10→10
*T*_min_ = 0.481, *T*_max_ = 0.565	*k* = −10→10
11063 measured reflections	*l* = −24→24

### Refinement

**Table:**

Refinement on *F*^2^	Secondary atom site location: difference Fourier map
Least-squares matrix: full	Hydrogen site location: inferred from neighbouring sites
*R*[*F*^2^ > 2σ(*F*^2^)] = 0.021	H-atom parameters constrained
*wR*(*F*^2^) = 0.045	*w* = 1/[σ^2^(*F*_o_^2^) + (0.0101*P*)^2^ + 0.1*P*] where *P* = (*F*_o_^2^ + 2*F*_c_^2^)/3
*S* = 1.06	(Δ/σ)_max_ = 0.001
2902 reflections	Δρ_max_ = 1.08 e Å^−^^3^
163 parameters	Δρ_min_ = −0.68 e Å^−^^3^
0 restraints	Absolute structure: Flack (1983), with 1206 Friedel pairs
Primary atom site location: structure-invariant direct methods	Flack parameter: 0.009 (7)

### Special details

**Table:**

Geometry. All s.u.'s (except the s.u. in the dihedral angle between two l.s. planes) are estimated using the full covariance matrix. The cell s.u.'s are taken into account individually in the estimation of s.u.'s in distances, angles and torsion angles; correlations between s.u.'s in cell parameters are only used when they are defined by crystal symmetry. An approximate (isotropic) treatment of cell s.u.'s is used for estimating s.u.'s involving l.s. planes.
Refinement. Refinement of *F*^2^ against ALL reflections. The weighted *R*–factor *wR* and goodness of fit *S* are based on *F*^2^, conventional *R*–factors *R* are based on *F*, with *F* set to zero for negative *F*^2^. The threshold expression of *F*^2^ > σ(*F*^2^) is used only for calculating *R*–factors(gt) *etc*. and is not relevant to the choice of reflections for refinement. *R*–factors based on *F*^2^ are statistically about twice as large as those based on *F*, and *R*–factors based on ALL data will be even larger.

### Fractional atomic coordinates and isotropic or equivalent isotropic displacement parameters (Å^2^)

**Table:**

	*x*	*y*	*z*	*U*_iso_*/*U*_eq_	
Hg1	0.26613 (2)	0.88923 (2)	0.290676 (8)	0.03034 (6)	
Cl1	0.29943 (16)	1.16949 (14)	0.31668 (6)	0.0395 (3)	
Cl2	0.35752 (15)	0.69726 (14)	0.37081 (6)	0.0377 (3)	
C1	−0.0295 (7)	0.8653 (6)	0.4070 (3)	0.0438 (14)	
H1A	0.0532	0.8345	0.4362	0.053*	
C2	−0.1777 (7)	0.8749 (6)	0.4328 (3)	0.0492 (15)	
H2A	−0.1975	0.8543	0.4794	0.059*	
C3	−0.2978 (7)	0.9151 (7)	0.3897 (3)	0.0567 (17)	
H3A	−0.4025	0.9204	0.4059	0.068*	
C4	−0.2644 (7)	0.9477 (6)	0.3223 (3)	0.0473 (13)	
H4A	−0.3461	0.9749	0.2918	0.057*	
C5	−0.1117 (6)	0.9401 (5)	0.3000 (2)	0.0322 (11)	
C6	−0.0662 (6)	0.9816 (6)	0.2286 (2)	0.0384 (13)	
H6A	−0.1586	0.9713	0.1985	0.046*	
H6B	−0.0320	1.0888	0.2274	0.046*	
C7	0.1322 (6)	0.9362 (5)	0.1386 (2)	0.0282 (11)	
H7A	0.1457	1.0486	0.1412	0.034*	
C8	0.0268 (6)	0.9017 (7)	0.0780 (2)	0.0379 (12)	
H8A	−0.0722	0.9592	0.0829	0.045*	
H8B	0.0005	0.7924	0.0782	0.045*	
C9	0.1023 (7)	0.9418 (6)	0.0101 (2)	0.0433 (14)	
H9A	0.0328	0.9087	−0.0273	0.052*	
H9B	0.1146	1.0532	0.0069	0.052*	
C10	0.2604 (6)	0.8673 (5)	0.0025 (2)	0.0418 (12)	
H10A	0.3092	0.9004	−0.0407	0.050*	
H10B	0.2469	0.7559	0.0006	0.050*	
C11	0.3670 (6)	0.9082 (7)	0.0615 (2)	0.0379 (13)	
H11A	0.3895	1.0182	0.0604	0.046*	
H11B	0.4676	0.8533	0.0569	0.046*	
C12	0.2906 (5)	0.8671 (5)	0.1300 (2)	0.0211 (10)	
H12A	0.2757	0.7547	0.1301	0.025*	
N1	0.0037 (5)	0.8977 (5)	0.34230 (19)	0.0347 (10)	
N2	0.0605 (4)	0.8849 (5)	0.20324 (18)	0.0277 (8)	
H2B	0.0364	0.7922	0.1924	0.033*	
N3	0.3965 (4)	0.9033 (6)	0.18723 (18)	0.0312 (10)	
H3B	0.4219	0.9987	0.1891	0.037*	
H3C	0.4701	0.8351	0.1867	0.037*	

### Atomic displacement parameters (Å^2^)

**Table:**

	*U*^11^	*U*^22^	*U*^33^	*U*^12^	*U*^13^	*U*^23^
Hg1	0.02454 (9)	0.03805 (10)	0.02844 (9)	0.00119 (9)	−0.00319 (9)	0.00272 (8)
Cl1	0.0418 (8)	0.0354 (6)	0.0412 (6)	−0.0061 (6)	0.0044 (5)	0.0003 (5)
Cl2	0.0344 (7)	0.0393 (7)	0.0393 (6)	0.0026 (6)	−0.0101 (6)	0.0055 (6)
C1	0.048 (3)	0.044 (4)	0.039 (3)	−0.004 (3)	0.010 (3)	0.000 (3)
C2	0.060 (4)	0.041 (3)	0.047 (3)	−0.014 (3)	0.023 (3)	−0.015 (3)
C3	0.044 (4)	0.054 (4)	0.072 (4)	−0.019 (3)	0.030 (3)	−0.026 (3)
C4	0.026 (3)	0.063 (3)	0.053 (3)	−0.001 (3)	0.000 (3)	−0.023 (3)
C5	0.024 (2)	0.031 (3)	0.042 (3)	−0.0020 (19)	−0.001 (2)	−0.009 (2)
C6	0.029 (3)	0.035 (3)	0.051 (3)	0.009 (2)	−0.006 (2)	−0.006 (2)
C7	0.031 (3)	0.027 (3)	0.026 (2)	−0.002 (2)	−0.003 (2)	0.0041 (19)
C8	0.038 (3)	0.042 (3)	0.035 (3)	0.005 (3)	−0.013 (2)	−0.002 (3)
C9	0.057 (4)	0.043 (3)	0.030 (3)	0.002 (3)	−0.011 (3)	0.002 (2)
C10	0.049 (3)	0.046 (3)	0.031 (2)	−0.003 (3)	0.002 (2)	−0.003 (2)
C11	0.039 (3)	0.043 (3)	0.031 (3)	0.000 (3)	0.006 (2)	−0.003 (2)
C12	0.024 (2)	0.022 (2)	0.0181 (18)	−0.0105 (19)	−0.0011 (18)	0.0004 (17)
N1	0.026 (2)	0.040 (3)	0.038 (2)	−0.005 (2)	0.0040 (18)	−0.001 (2)
N2	0.0269 (19)	0.025 (2)	0.031 (2)	0.0037 (17)	−0.0025 (17)	−0.001 (2)
N3	0.026 (2)	0.035 (2)	0.033 (2)	0.001 (2)	−0.0007 (16)	−0.0011 (19)

### Geometric parameters (Å, º)

**Table:**

Hg1—N3	2.324 (4)	C7—C12	1.492 (6)
Hg1—Cl2	2.4426 (12)	C7—C8	1.524 (6)
Hg1—N2	2.458 (3)	C7—H7A	1.0000
Hg1—N1	2.460 (4)	C8—C9	1.525 (7)
Hg1—Cl1	2.5415 (13)	C8—H8A	0.9900
C1—N1	1.336 (6)	C8—H8B	0.9900
C1—C2	1.364 (8)	C9—C10	1.508 (7)
C1—H1A	0.9500	C9—H9A	0.9900
C2—C3	1.378 (9)	C9—H9B	0.9900
C2—H2A	0.9500	C10—C11	1.519 (7)
C3—C4	1.387 (8)	C10—H10A	0.9900
C3—H3A	0.9500	C10—H10B	0.9900
C4—C5	1.376 (7)	C11—C12	1.541 (6)
C4—H4A	0.9500	C11—H11A	0.9900
C5—N1	1.342 (6)	C11—H11B	0.9900
C5—C6	1.505 (7)	C12—N3	1.479 (5)
C6—N2	1.465 (6)	C12—H12A	1.0000
C6—H6A	0.9900	N2—H2B	0.8700
C6—H6B	0.9900	N3—H3B	0.8700
C7—N2	1.483 (6)	N3—H3C	0.8700
			
N3—Hg1—Cl2	116.76 (12)	C9—C8—H8B	109.0
N3—Hg1—N2	74.25 (13)	H8A—C8—H8B	107.8
Cl2—Hg1—N2	132.01 (10)	C10—C9—C8	111.3 (4)
N3—Hg1—N1	142.75 (13)	C10—C9—H9A	109.4
Cl2—Hg1—N1	92.57 (11)	C8—C9—H9A	109.4
N2—Hg1—N1	68.91 (12)	C10—C9—H9B	109.4
N3—Hg1—Cl1	94.08 (13)	C8—C9—H9B	109.4
Cl2—Hg1—Cl1	120.60 (4)	H9A—C9—H9B	108.0
N2—Hg1—Cl1	103.66 (10)	C9—C10—C11	110.8 (4)
N1—Hg1—Cl1	89.37 (11)	C9—C10—H10A	109.5
N1—C1—C2	122.5 (6)	C11—C10—H10A	109.5
N1—C1—H1A	118.7	C9—C10—H10B	109.5
C2—C1—H1A	118.7	C11—C10—H10B	109.5
C1—C2—C3	118.4 (5)	H10A—C10—H10B	108.1
C1—C2—H2A	120.8	C10—C11—C12	111.1 (4)
C3—C2—H2A	120.8	C10—C11—H11A	109.4
C2—C3—C4	119.3 (5)	C12—C11—H11A	109.4
C2—C3—H3A	120.3	C10—C11—H11B	109.4
C4—C3—H3A	120.3	C12—C11—H11B	109.4
C5—C4—C3	119.3 (6)	H11A—C11—H11B	108.0
C5—C4—H4A	120.4	N3—C12—C7	112.2 (3)
C3—C4—H4A	120.4	N3—C12—C11	111.0 (3)
N1—C5—C4	120.7 (5)	C7—C12—C11	112.7 (4)
N1—C5—C6	117.3 (4)	N3—C12—H12A	106.8
C4—C5—C6	122.0 (5)	C7—C12—H12A	106.8
N2—C6—C5	111.5 (4)	C11—C12—H12A	106.8
N2—C6—H6A	109.3	C1—N1—C5	119.7 (5)
C5—C6—H6A	109.3	C1—N1—Hg1	125.5 (4)
N2—C6—H6B	109.3	C5—N1—Hg1	114.8 (3)
C5—C6—H6B	109.3	C6—N2—C7	114.7 (4)
H6A—C6—H6B	108.0	C6—N2—Hg1	106.2 (3)
N2—C7—C12	110.3 (3)	C7—N2—Hg1	107.6 (3)
N2—C7—C8	111.6 (4)	C6—N2—H2B	117.2
C12—C7—C8	111.3 (4)	C7—N2—H2B	100.0
N2—C7—H7A	107.8	Hg1—N2—H2B	110.9
C12—C7—H7A	107.8	C12—N3—Hg1	111.3 (3)
C8—C7—H7A	107.8	C12—N3—H3B	113.1
C7—C8—C9	113.0 (4)	Hg1—N3—H3B	97.7
C7—C8—H8A	109.0	C12—N3—H3C	106.4
C9—C8—H8A	109.0	Hg1—N3—H3C	108.7
C7—C8—H8B	109.0	H3B—N3—H3C	119.3

### Hydrogen-bond geometry (Å, º)

**Table:**

*D*—H···*A*	*D*—H	H···*A*	*D*···*A*	*D*—H···*A*
N3—H3*B*···Cl2^i^	0.87	2.83	3.527 (5)	138
N3—H3*C*···Cl1^ii^	0.87	2.45	3.316 (5)	173

Symmetry codes: (i) −*x*+1, *y*+1/2, −*z*+1/2; (ii) −*x*+1, *y*−1/2, −*z*+1/2.
